# Association of common polymorphisms in the *IL2RA* gene with type 1 diabetes: evidence of 32,646 individuals from 10 independent studies

**DOI:** 10.1111/jcmm.12642

**Published:** 2015-08-07

**Authors:** Wei Tang, Dai Cui, Lin Jiang, Lijuan Zhao, Wei Qian, Sarah Alice Long, Kuanfeng Xu

**Affiliations:** a The Affiliated Jiangyin Hospital of Southeast University Medical CollegeJiangyin, Jiangsu, China; b Department of Endocrinology, The First Affiliated Hospital of Nanjing Medical UniversityNanjing, China; c Translational Research Program, Benaroya Research Institute at Virginia MasonSeattle, WA, USA

**Keywords:** interleukin 2 receptor alpha, single nucleotide polymorphism, type 1 diabetes, meta-analysis

## Abstract

Single nucleotide polymorphisms (SNPs) in the interleukin 2 receptor alpha (*IL2RA*) gene have been suggested to be associated with type 1 diabetes (T1D) susceptibility. However, the results from individual studies are inconsistent. To explore the association of *IL2RA* polymorphisms with T1D, including rs11594656, rs2104286, rs3118470, rs41295061 and rs706778, a meta-analysis involving 10 independent studies with 19 outcomes was conducted: five studies with a total of 10,572 cases and 12,956 controls were analysed for rs11594656 with T1D risk, three studies with 7300 cases and 8331 controls for rs2104286, three studies with 3880 cases and 5409 controls for rs3118470, five studies with 11,253 cases and 13,834 controls for rs41295061 and three studies with 1896 cases and 1709 controls for rs706778 respectively. Using minor allelic comparison, the five investigated SNPs were all observed to have a significant association with T1D: For rs11594656, fixed effect model (FEM) odds ratio (OR) 0.87, 95% confidence interval (CI) 0.83, 0.91; rs2104286, FEM OR 0.81, 95% CI 0.77, 0.85; rs3118470, FEM OR 1.23, 95% CI 1.16, 1.31; rs41295061, random effect model (REM) OR 0.67, 95% CI 0.60, 0.76 and rs706778 FEM OR 1.20, 95% CI 1.08, 1.33. Similar results were obtained when all the included studies were calculated by a REM. Our meta-analysis suggests that all five SNPs in the *IL2RA* gene are risk factors for T1D risk, and rs11594656, rs2104286 and rs41295061 are the most associated SNPs in the populations investigated. This conclusion warrants confirmation by further studies.

## Introduction

Type 1 diabetes (T1D) is a complex, multigenetic autoimmune disease featured by destruction of the insulin producing beta-cells of the pancreas by autoreactive T lymphocytes. The interleukin 2 receptor alpha (*IL2RA*, also known as *CD25*) encodes the α-chain of the IL-2 receptor complex and binds to IL-2 with high-affinity. Studies have indicated that *IL-2/IL-2RA*-mediated regulatory mechanisms play a central role in preventing T1D 1. Furthermore, an *IL-2/IL-2RA*-dependent proliferation of CD4^+^ FOXP3^+^ Tregs could be strongly linked to their efficiency in the immune homoeostasis, as impairment of CD4^+^ FOXP3^+^ Treg in T1D occurs primarily because of the inefficient induction and maintenance of Tregs, with deficiencies in *IL-2/IL2RA* signalling 2,3.

The association of the *IL2RA* locus with T1D was first investigated by Vella *et al*. 4 using a multilocus tag single nucleotide polymorphism (SNP) approach, and subsequently replicated in three genome-wide association studies (GWAS), including WTCCC, GoKinD and NIMH, and several other case-control studies in T1D. Five common tag SNPs were mainly observed, which had no obvious linkage disequilibrium to each other 5–21. These are two intronic SNPs in the 5′ region of *IL2RA*, rs706778 and rs3118470; two SNPs mapping to the 5′ flanking region, rs41295061 (previously named ss52580101 or rs12722495) and rs11594656; and one SNP in the intron 1, rs2104286. Furthermore, it has been demonstrated that rs41295061 was associated with glutamate decarboxylase antibody positivity in T1D patients 22, and Lowe *et al*. 5 found that rs11594656, rs2104286 and rs41295061 independently correlated with the circulating concentration of soluble form of IL2RA. These SNPs may present independent biological pathways that contribute to disease susceptibility, including transcriptional regulation of IL2RA and levels of surface expression of IL-2RA.

Despite strong functional evidence for the correlation of these SNPs with the immune status in T1D, the results of the genetic association studies of T1D remain inconsistent. To evaluate the potential role of these five SNPs in influencing T1D susceptibility, we performed a meta-analysis on eligible studies to confirm the association.

## Subjects and methods

### Search strategy

Electronic databases (Medline and EMBASE) were searched up to March 2015 for all genetic association studies evaluating the *IL2RA* gene polymorphisms in T1D. Search strategies were investigated using combinations of the following search terms: (*IL2RA* or *CD25*) and (T1D) and (variant or allele or polymorphism). The publication language was restricted to English, but no restriction was set on the source of control participants (general population, clinic or hospital). To identify additional relevant studies, we also searched the reference lists and the Medline option ‘Related Articles’ of the selected articles.

### Inclusion criteria and data extraction

Any human genetic association study, regardless of sample size, was included in the meta-analysis if it met the following criteria: (*i*) study evaluated the association of *IL2RA* polymorphisms (rs11594656 and/or rs2104286 and/or rs3118470 and/or rs41295061 and/or rs706778) with T1D; (*ii*) study had sufficient published data to estimate an odds ratio (OR) with 95% confidence interval (CI) or provided raw data that allowed us to calculate them; (*iii*) if the data were duplicated or had been published more than once, the most recent and complete study was chosen; (*iv*) studies were excluded if the genotype distribution of the controls deviated from Hardy-Weinberg equilibrium (HWE) and (*v*) review articles, abstracts, editorials, reports with incomplete data and studies based on pedigree data were also excluded.

Information extracted from each study was considered as follows: name of first author, publication year, ethnic origin, number of participants in cases and controls, genotype and allele frequency by case–control status and OR (95% CI). Not all articles reported the necessary statistics directly, so in some instances we transformed and estimated an OR from the reported data 23. All the identified studies were carefully reviewed by two investigators independently, and any discrepancies were resolved by discussion, when necessary, adjudicated by a third reviewer. All participants of the included studies provided informed consent and the studies were approved by the ethics committees of the participating institutions.

### Statistical analysis

The distribution was considered to be deviated from HWE at *P* < 0.05 for case-control studies and *P* < 10^−5^ for GWAS 24. For each SNP where data were available from at least three studies, a meta-analysis was carried out as described previously 25. Pooled ORs with 95% CI were used to assess the strength of association in minor allelic risk. The significance of the pooled OR was determined by the *Z*-test, and *P* < 0.05 was considered statistically significant. The heterogeneity between the studies was evaluated with chi-squared based Q statistic and *I*^2^ metric. Heterogeneity was considered significant at *P* < 0.05 for the Q statistic and *I*^2^ > 50% for the *I*^2^ metric. The pooled OR was calculated by a fixed effect model (FEM; using the Mantel-Haenszel method) or a random effect model (REM; using the DerSimonian-Laird method) according to the heterogeneity among studies 26,27. To evaluate the stability of the results, sensitivity and influence analysis were performed. Further to address the issue of false-positive association of each SNP, the false-positive report probability (FPRP) test of Wacholder *et al*. 28 was also performed. Publication bias was assessed by modified Begg’s test and Egger’stest (*P* < 0.05 was considered statistically significant). All statistical analyses were conducted using STATA version 11.0 (Stata, College Station, TX, USA).

## Results

### Characteristics of study

**Figure 1:**
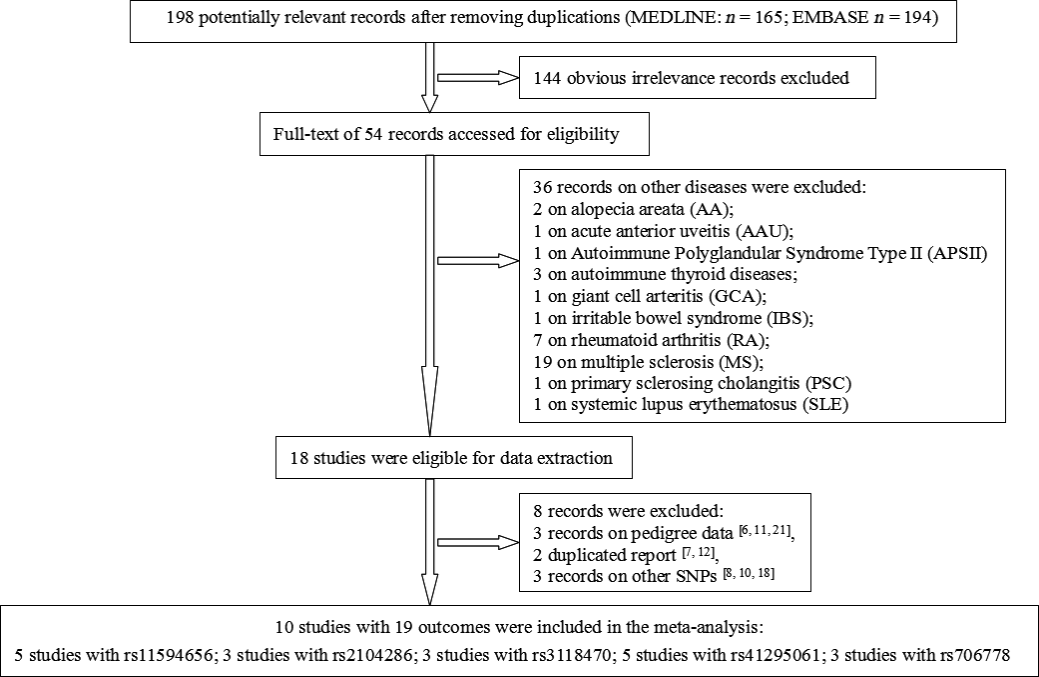
Flow chart of meta-analysis for selecting published studies on the association of IL2RA polymorphism and T1D risk.

A total of 10 studies 5,9,13–17,19,20 with 19 outcomes met the inclusion and exclusion criteria (Fig. 1). All were case-control studies and most were population-based. Of these, eight studies involved Europeans, two were of Asians. The genotype frequency in controls was in HWE for all included studies. Some studies only provided ORs with 95% CIs under the minor allelic comparison, hence the summary estimate was calculated with this model. Their characteristics are listed (Table 1). The association of rs11594656, rs2104286, rs3118470, rs41295061 and rs706778 polymorphisms with T1D was examined in 5, 3, 3, 5 and 3 studies respectively.

**Table 1:** Allelic and genotype distributions of the IL2RA polymorphisms for T1D risk in studies included in the meta-analysis

Authors [ref.]	Year	Country	Ethnicity	Total/Genotypes (11/12/22)		MAF (%)		OR (95% CI)	SNPs
				Cases	Controls	Cases	Controls		
Lowe *et al*. -set 1 5	2007	UK	European	2965 (1744/994/136)	2548 (1385/956/143)	0.213	0.244	0.84 (0.76–0.92)	rs11594656
Lowe *et al*. -set 2 5	2007	UK	European	5259 (3186/1827/246)	6809 (3850/2548/411)	0.220	0.247	0.87 (0.81–0.92)	rs11594656
Lowe *et al*. -set 1 5	2007	UK	European	2965 (2543/344/20)	2548 (2002/457/35)	0.065	0.103	0.61 (0.53–0.70)	rs41295061
Lowe *et al*. -set 2 5	2007	UK	European	5312 (4609/675/28)	6855 (5520/1250/85)	0.069	0.104	0.65 (0.59–0.71)	rs41295061
Kawasaki *et al*. 9	2009	Japan	Asian	882 (836/43/2)	606 (570/35/1)	0.027	0.031	0.91 (0.59–1.41)*	rs11594656
Kawasaki *et al*. 9	2009	Japan	Asian	872 (206/427/239)	592 (159/298/135)	0.519	0.479	1.18 (1.01–1.35)*	rs3118470
Kawasaki *et al*. 9	2009	Japan	Asian	877 (307/421/149)	602 (170/309/123)	0.410	0.461	1.23 (1.06–1.43)*	rs706778
Maier *et al*. 12	2009	UK/US	European	6425 (ND)	6862 (ND)	ND	ND	0.80 (0.76–0.85)	rs2104286
Grant *et al*. -set 1 13	2009	UK	European	2000 (ND)	3000 (ND)	0.361	0.319	1.21 (1.11–1.31)*	rs3118470
Grant *et al*. -set 2 13	2009	US	European	563 (ND)	1146 (ND)	0.365	0.306	1.30 (1.12–1.52)*	rs3118470
Aminkeng *et al*. 14	2010	Belgium	European	1954 (ND)	2082 (ND)	0.054	0.084	0.63 (0.52–0.75)	rs41295061
Klinker *et al*. 15	2010	Finland	European	591 (ND)	1538 (ND)	ND	ND	0.98 (0.82–1.17)	rs11594656
Klinker *et al*. 15	2010	Finland	European	591 (ND)	1538 (ND)	ND	ND	0.95 (0.74–1.25)*	rs41295061
Yamashita *et al*. 16	2011	Japan	Asian	790 (ND)	953 (ND)	ND	ND	1.2 (1.0–1.4)	rs706778
Espino-Paisán *et al*. 17	2011	Spain	European	430 (205/179/46)	791 (375/330/86)	0.315	0.317	0.99 (0.82–1.19)	rs11594656
Espino-Paisán *et al*. 17	2011	Spain	European	430 (277/135/18)	798 (488/268/42)	0.199	0.221	0.88 (0.71–1.08)	rs2104286
Espino-Paisán *et al*. 17	2011	Spain	European	431 (393/35/3)	811 (704/105/2)	0.048	0.067	0.69 (0.47–1.02)	rs41295061
Kisand *et al*. 19	2012	Estonia	European	229 (ND)	154 (ND)	0.47	0.45	1.08 (0.81–1.44)*	rs706778
Fichna *et al*. 20	2012	Poland	European	445 (273/155/17)	671 (373/248/50)	0.212	0.259	0.77 (0.63–0.94)*	rs11594656
Fichna *et al*. 20	2012	Poland	European	445 (312/123/10)	671 (457/187/27)	0.161	0.180	0.89 (0.72–1.09)*	rs2104286
Fichna *et al*. 20	2012	Poland	European	445 (153/217/75)	671 (283/306/72)	0.412	0.335	1.30 (1.09–1.55)*	rs3118470

* OR and 95% CI were calculated under the minor allelic comparison from the reported genotypes or minor allele frequency.

11 Homozygous for major allele, 12 heterozygous, 22 homozygous for minor allele.

ND: no data (no genotype data available); MAF: minor allele frequency; SNP: single nucleotide polymorphisms.

### Quantitative synthesis

Results of pooled analyses are summarized in detail (Table 2 and Figs 6). Our meta-analysis all showed significant overall associations between the 5 investigated SNPs and T1D risk: For rs11594656, FEM OR 0.87, 95% CI 0.83, 0.91; rs2104286, FEM OR 0.81, 95% CI 0.77, 0.85; rs3118470, FEM OR 1.23, 95% CI 1.16, 1.31; rs41295061, REM OR 0.67, 95% CI 0.60, 0.76 and rs706778 FEM OR 1.20, 95% CI 1.08, 1.33. Moreover, the results were similar when all the included studies were calculated by a REM (Fig. S1).

**Table 2:** Pooled measures for the association between the IL2RA gene polymorphisms and susceptibility to T1D

SNPs	*n* *			Heterogeneity		Model	OR (95% CI)†	*P* ‡
	Studies	Cases	Controls	*I*^2^ (%)	*P*			
rs11594656	5	10,572	12,956	10.0	0.352	FEM	0.87 (0.83–0.91)	<10^−6^
rs2104286	3	7300	8331	0	0.453	FEM	0.81 (0.77–0.85)	<10^−6^
rs3118470	3	3880	5409	0	0.718	FEM	1.23 (1.16–1.31)	<10^−6^
rs41295061	5	11,253	13,834	55.6	0.061	REM	0.67 (0.60–0.76)	<10^−6^
rs706778	3	1896	1709	0	0.734	FEM	1.20 (1.08–1.33)	0.001

* Number of studies included.

† Odds ratio with 95% confidential interval for pooled effect size.

‡ Significance of pooled effect size.

FEM: fixed effect model; REM: random effect model; SNP: single nucleotide polymorphisms.

**Figure 2:**
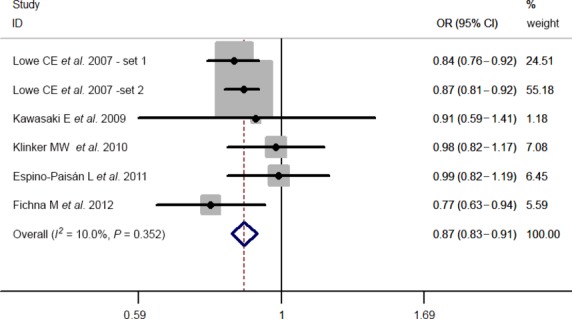
Stratified analysis pooled ORs for the association between rs11594656 and susceptibility to T1D. The area of the squares reflects the study-specific weight. The diamond shows the summary random-effects OR estimate.

**Figure 3:**
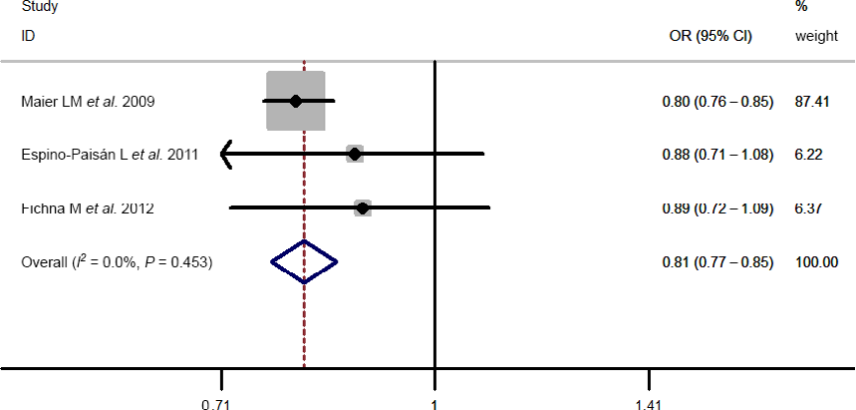
Stratified analysis pooled ORs for the association between rs2104286 and susceptibility to T1D. The area of the squares reflects the study-specific weight. The diamond shows the summary random-effects OR estimate.

**Figure 4:**
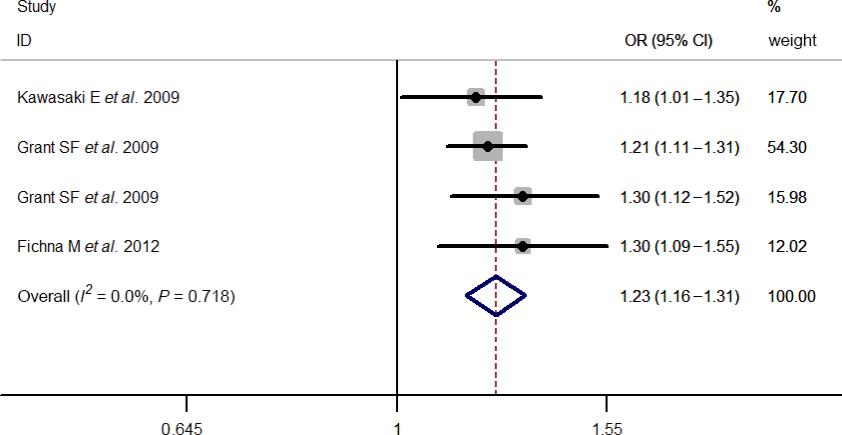
Stratified analysis pooled ORs for the association between rs3118470 and susceptibility to T1D. The area of the squares reflects the study-specific weight. The diamond shows the summary random-effects OR estimate.

**Figure 5:**
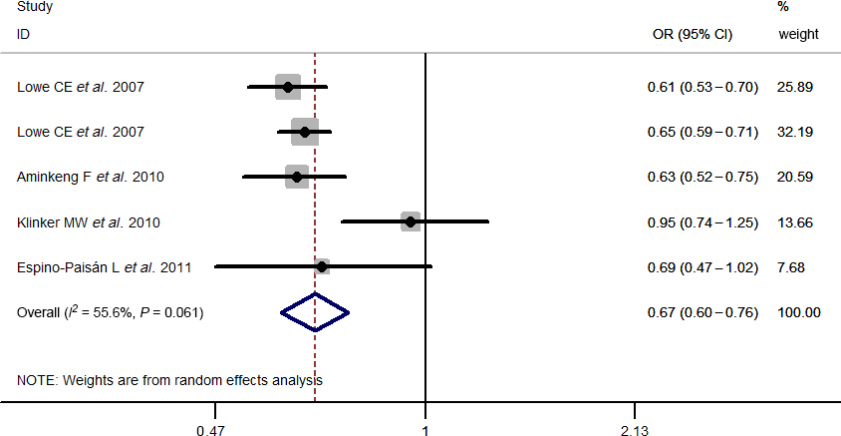
Stratified analysis pooled ORs for the association between rs41295061 and susceptibility to T1D. The area of the squares reflects the study-specific weight. The diamond shows the summary random-effects OR estimate.

**Figure 6:**
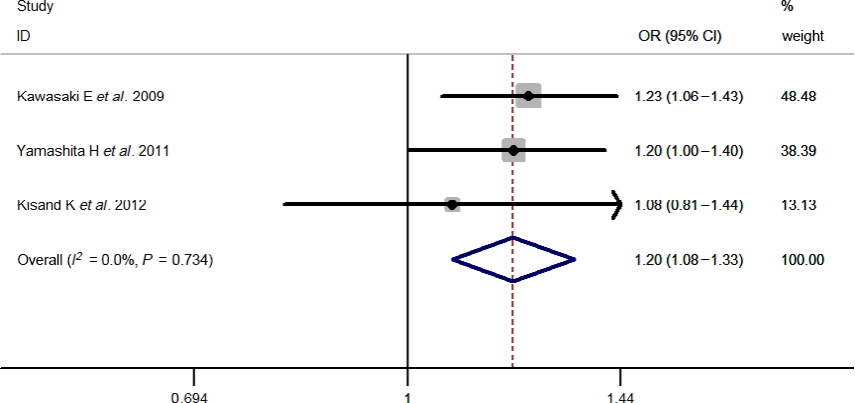
Stratified analysis pooled ORs for the association between rs706778 and susceptibility to T1D. The area of the squares reflects the study-specific weight. The diamond shows the summary random-effects OR estimate.

### Heterogeneity and influence analysis

Significant heterogeneity was only observed in the studies investigating rs41295061 polymorphism (Table 2), and sensitivity analysis was conducted. When omitting Klinker *et al*. 15, the association was also significantly observed, but the heterogeneity was effectively eliminated, which indicated this study was mainly responsible for the observed heterogeneity (Fig. S2). Furthermore, to assess the degree to which each individual study affected the overall OR estimates, influence analysis was conducted by repeating the meta-analysis sequentially excluding one study at a time. The results indicated no single study excessively influenced the analysis, except for Maier *et al*. 12, of rs2104286 polymorphisms (changing to FEM OR 0.89, 95% CI 0.76, 1.03) (Fig. S3).

### Publication bias

As expected, the funnel plots for the associations of the investigated SNPs with T1D were symmetrical and the results for modified Begg’s and Egger’s tests were not significant, confirming that our results were statistically robust and not affected by publication bias (Table S1).

## Discussion

Several GWASs and a number of case-control studies have examined the association between the investigated five SNPs and T1D risk, but the results showed significant between-study variation. So we conducted a meta-analysis to obtain a more definitive conclusion. Our meta-analysis results suggested all the investigated SNPs had a significant association with T1D risk in overall. Although the influence analysis indicated that the study from Maier *et al*. 12 excessively influenced the association of rs2104286 polymorphism, the FPRP value of this SNP suggested a <20% chance of the result being a false positive when assigned a high prior probability range (*i.e*. 0.00001–0.0001; data not shown), which implicated that the results may be statistically robust.

The five investigated SNPs were independently tagged polymorphisms of IL2RA gene. Studies have indicated that the rs41295061 protective haplotype that is only associated with T1D, the rs2104286 protective haplotype that is associated with MS, T1D and RA and the rs11594656 haplotype that is associated with protection from T1D but risk for MS 5,12,29. A study from Qu *et al*. 6 also found that rs706778 and rs3118470 exhibited highly significant association with T1D independently. Further studies indicated that their biological functions were quite different. A study from Belot *et al*. 30 indicated that rs11594656, rs41295061 and rs2104286 had a strong association with the methylation of CpG -373, but their contributions to the variance of methylation at CpG -373 were different. Another study indicated differential, allele-specific binding of the transcription factors CREB and TFAP4 to IL2RA SNPs rs41295061*A and rs2104286*A 31. Furthermore, Cerosaletti *et al*. 32 found decreased pSTAT5 and increased CD25 expression on naïve Treg in cases carrying the rs2104286 risk haplotype. As for the other two SNPs, rs3118470 and rs706778, they were found to be highly acetylated in T cells and involved in indirectly disrupting IL-2RA transcription 33. Taken together, these indeed suggested for the role of three *IL2RA* locus, rs11594656, rs2104286 and rs41295061, as a general autoimmunity gene contributing to the pathogenesis of autoimmune diseases, such as T1D. And the other two SNPs need to be further verified by a greater number of participants.

Heterogeneity is potentially a significant problem when interpreting the results of any meta-analysis of genetic association studies 34. Our meta-analysis showed significant between-study heterogeneity only existed in rs41295061 polymorphism. Many of the variables that varied between different studies might be responsible for this observed heterogeneity, including the source of the controls, sex bias, age, *etc*. Initial inspection of the data did not immediately identify any likely candidate variable or study that was significantly impacting on the result. The reason for this is unclear, but it may be that populations also have environmental differences that affect their sensitivity to particular genomic variants, such as viral infections, toxins and diet.

The current meta-analysis should also be interpreted within the context of a number of limitations. Only two studies from Asian descendents were included in the meta-analysis 9,16, and their sample size was relatively small, therefore further stratified analysis by ethnicity could not be performed, although studies have indicated the difference of susceptibility genotypes between Europeans and Asians 25,35. This suggested more additional well-powered studies from Asians should be performed to confirm the association, involving large population and family collections. Similarly, besides ethnicity, other potential environment × gene interactions may well be contributors to the observed disease-effect unconformity, but we had insufficient data to perform an evaluation of such interactions. Furthermore, we thoroughly investigated heterogeneity and study-size effects and estimated *IL2RA* polymorphisms. However, we could not assess the haplotype effects, which would require individual patient data or compound genotype summary data. In addition, other SNPs in the *IL2RA* gene were also reported to be associated with T1D, such as rs4147359 5,6 and rs61839660 18. Yet, there are no insufficient studies to perform a meta-analysis to confirm this association.

In conclusion, our results suggest that the five investigated SNPs in the *IL2RA* gene are significantly associated with T1D independently. We suggest that additional larger studies allowing stratification for other gene × environment interactions should be performed to further clarify the possible roles of these genetic variants in the aetiology of T1D.

## Supporting Information

**Figure S1** Stratified analysis pooled ORs for the association between the five SNPs of IL2RA gene and susceptibility to T1D in a random effect model. The area of the squares reflects the study-specific weight. The diamond shows the summary random-effects OR estimate.

**Figure S2** Pooled ORs for the association between rs41295061 polymorphism and T1D when omitting the study of Klinker *et al*. The area of the squares reflects the study-specific weight. The diamond shows the summary random-effects OR estimate.

**Figure S3** Influence analysis on the assocaition between rs2104286 and susceptibility to T1D by repeating sequentially excluding one study at a time.

**Table S1** Egger’s publication bias test for the rs11594656, rs2104286, rs3118470, rs41295061 and rs706778 polymorphisms in T1D risk.
