# Supplementary figures and images for: Association of common polymorphisms in the IL2RA gene with type 1 diabetes: evidence of 32,646 individuals from 10 independent studies
Source: J Cell Mol Med. 2015 Aug 7;19(10):2481–8. doi: 10.1111/jcmm.12642 (PMC4594689; doi:10.1111/jcmm.12642)

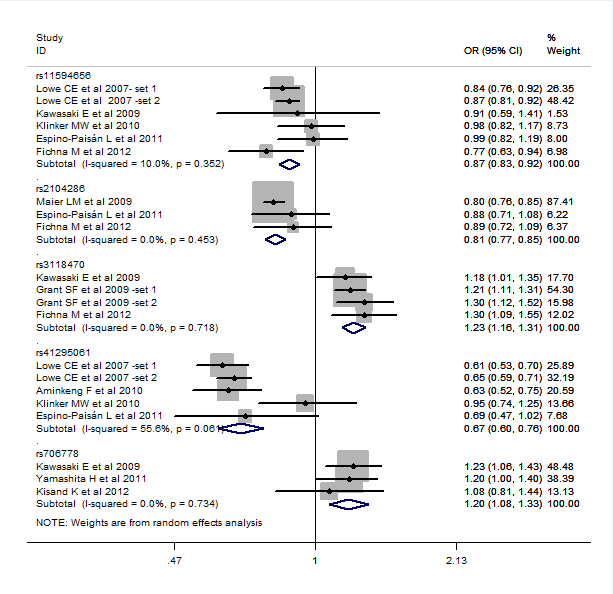

Supplement: Supplementary file 1 [file jcmm0019-2481-sd1.tif]

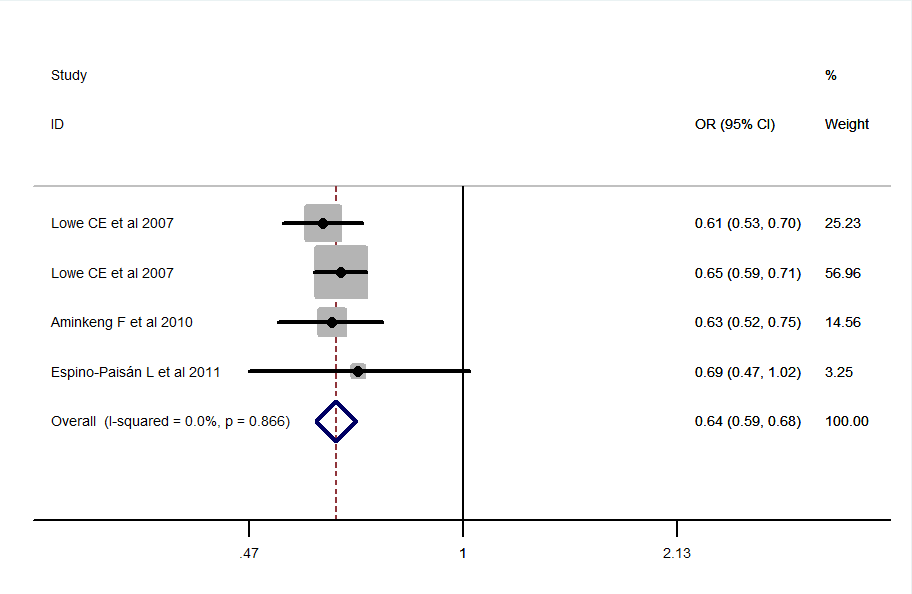

Supplement: Supplementary file 2 [file jcmm0019-2481-sd2.tif]

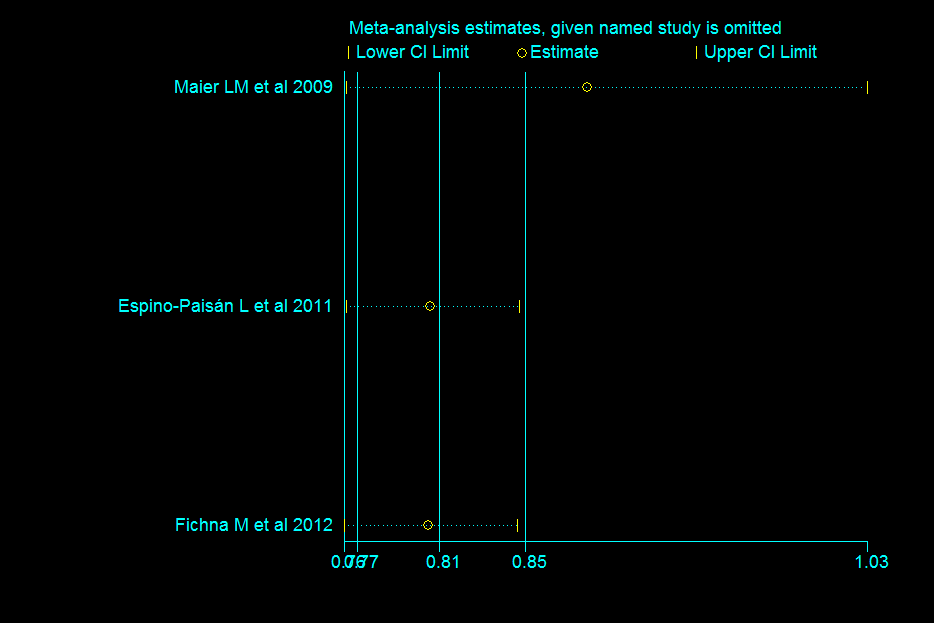

Supplement: Supplementary file 3 [file jcmm0019-2481-sd3.tif]
