# Supplementary material for: Association of common polymorphisms in the IL2RA gene with type 1 diabetes: evidence of 32,646 individuals from 10 independent studies
Source: J Cell Mol Med. 2015 Aug 7;19(10):2481–8. doi: 10.1111/jcmm.12642 (PMC4594689; doi:10.1111/jcmm.12642)
Supplement: Supplementary file 4 [file jcmm0019-2481-sd4.doc]

**Table S1 Egger’s publication bias test for the rs11594656, rs2104286, rs3118470, rs41295061 and rs706778 polymorphisms in T1D risk.**

| **SNPs** | **Coef.** | **Std. Err.** | **t** | **P>|t|** | **[95% Conf. Interval]** |
| --- | --- | --- | --- | --- | --- |
| rs11594656 | 0.505 | 0.904 | 0.56 | 0.606 | -2.005, 3.015 |
| rs2104286 | 1.297 | 0.088 | 14.66 | 0.043 | 0.173, 2.421 |
| rs3118470 | 1.132 | 1.077 | 1.05 | 0.404 | -3.502, 5.766 |
| rs41295061 | 1.549 | 1.548 | 1.00 | 0.391 | -3.376, 6.474 |
| rs706778 | -1.829 | 0.153 | -11.95 | 0.053 | -3.774, 0.116 |
